# Case Report: Nutritional Examination of Weight Loss Treatment Using Kampo

**DOI:** 10.3389/fnut.2021.551373

**Published:** 2021-01-27

**Authors:** Yutaka Matsui, Ikuko Matsui

**Affiliations:** Matsui Dietary & Dementia Clinic, Akashi, Japan

**Keywords:** muscle mass, weight loss, dementia, loss of appetite, Ninjin'yoeito

## Abstract

Loss of appetite is a common symptom in patients with dementia, and if weight loss or difficulty eating occurs without subjective symptoms, the patient can easily become malnourished. There is also a close relationship between dementia and physical frailty, such as weight loss and muscle weakness, and thus early intervention to address frailty in patients with dementia is important. In this study, 3 patients with dementia who complained of loss of appetite and weight loss showed increases in body weight and muscle mass after taking Ninjin'yoeito. Ninjin'yoeito was found to be a potentially effective treatment option for physical frailty in patients with dementia.

## Introduction

Loss of appetite is a common symptom in patients with dementia, and when weight loss or difficulty eating occur in the absence of subjective symptoms, the patient can easily become malnourished. In such cases, the underlying causes should be carefully investigated. If the loss of appetite is caused by an organic abnormality of the digestive system or worsening of neurological function (e.g., dysphagia, constipation) associated with extrapyramidal symptoms, the patient should be treated for the dysfunction. If these possible causes are excluded, energy supplementation should be provided along with dietary nutritional guidance and prescriptions of oral and enteral nutritional supplements. Selective serotonin reuptake inhibitors (SSRIs), serotonin and norepinephrine reuptake inhibitors (SNRIs), or second-generation antipsychotics (atypical antipsychotics) are used to treat loss of appetite caused by apathy and depression in dementia. Patients who do not respond to treatment may experience repeated episodes of malnutrition and dehydration, leading to heart failure, infection, and potentially fatal outcomes. Therefore, loss of appetite in the elderly and patients with dementia has a significant impact on their life prognosis and should not be overlooked.

In addition, elderly individuals with loss of appetite and patients with dementia who remain malnourished are prone to fall into the frailty cycle, in which their basal metabolism and activity level are decreased due to reduced muscle mass and strength, leading to further loss of appetite and weight loss. Frailty is a condition in which physical and mental vitality, such as motor and cognitive functions, declines with age, leading to functional impairment of activities of daily living (ADLs), and a state that requires long-term care. There is a close relationship between dementia and frailty whereby physical frailty, such as weight loss and muscle weakness, causes a person to be at risk for cognitive decline or dementia, and conversely, cognitive impairment causes a person to be at risk for physical frailty. It has been reported that as cognitive decline progresses, the level of frailty increases (1, 2). Therefore, early intervention to address frailty, including weight loss and muscle weakness, is important in patients with dementia. Our hospital is conducting active nutritional management, including a food frequency questionnaire, when loss of appetite or weight loss is a concern. However, in some patients nutritional status cannot be adequately controlled even with such guidance, in which case traditional Japanese herbal medicine (Kampo) may be introduced.

Ninjin'yoeito, Japanese herbal medicine (Kampo), is indicated for the treatment of loss of appetite and has been used since ancient times to improve nutritional status. In recent years, it has also been applied to treat frailty because of its multifaceted effects (3), and there have been numerous reports showing improvements in musculoskeletal strength and mass in addition to reversal of appetite loss, but none yet for muscle mass.

In this study, 3 cases of patients with dementia who experienced loss of appetite and weight loss, and who gained weight and muscle mass after receiving Ninjin'yoeito are reported. Informed written consent was obtained from the patients and their caregivers regarding this report.

## Case Descriptions

### Case 1

The patient was an 84-year-old female; 145.1 cm in height and 54.0 kg in weight at the time of her initial examination; a housewife; and described as sincere and calm in personality. Her chief complaints were loss of appetite and weight loss, and she had a medical history of angina with onset at the age of 75. With regard to the history of her present illness, she came to our hospital in January X−3 due to noticeable amnesia such as not being able to recognize the faces and names of relatives and grandchildren. She was diagnosed with Alzheimer's disease based on a 16-point score on the Mini-Mental State Examination (MMSE) and on functional imaging findings such as cerebral blood flow single-photon emission computed tomography (SPECT). She was treated with galantamine and outpatient care for lifestyle diseases such as hypertension, and her instrumental ADLs (IADLs) improved to the point where she was able to prepare meals, go shopping, and take the bus.

She was treated with galantamine. However, her functional ability to perform ADLs gradually declined; and in the summer of X−1, her functional impairment in ADLs progressed to the point where she required emergency treatment, including intravenous fluids, due to heat stroke. In December X−1, she fell on the stairs at a train station, fractured her right femur, and underwent an artificial femoral head replacement. Although she was discharged from the hospital in April X, she was unable to take her medication or do household tasks on her own that she was previously able to do. She was unable to prepare meals and often did not eat when her eldest daughter prepared meals for her. In October X, she had frequent hallucinations at home when someone was in the room; she started to eat much less food; and she weighed 50.4 kg, showing a decrease of 4 kg in 6 months. Although she was able to walk on her own, her IADLs declined as indicated by scores of 48 on the Dementia Assessment Sheet for Community-based Integrated Care System 21-items (DASC-21), 4 on the Lawton IADL scale, 3 on the level of long-term care required, 21 on the MMSE, and 14 on the Frontal Assessment Battery (FAB). Her muscle mass was 30.2 kg as measured by a body composition analyzer (InBody 270) using bioelectrical impedance analysis. The calculated weight after removing the body fluid weight (19.1 kg) from the total body weight (50.4 kg) was 31.3 kg. Since October X, she had been instructed to take 3.75 g of Ninjin'yoeito once before bedtime for her loss of appetite and weight loss.

In April X+1, improvement in her appetite was observed. Her weight was 52.2 kg, showing an increase of 1.8 kg. Her muscle mass was 31.1 kg, showing an increase of 0.9 kg. The weight after subtracting the body fluid weight was 52.2–19.7 kg = 32.5 kg, showing an increase of 1.2 kg. In July X+1, her weight dropped slightly by 0.8 to 51.4 kg, and her muscle mass also dropped by 0.3 to 30.8 kg. Eventually, there was an increase of 1.0 kg in body weight and 0.6 kg in muscle mass, and the weight after removing the body fluid weight was 51.4–19.5 kg = 31.9 kg, showing an increase of 0.6 kg. With regard to ADLs, a decrease in basic ADLs (BADLs) in addition to IADLs was observed, such as leaving a pot on the fire, requiring assistance to take medication, being unable to flush the toilet, and being unable to bathe and wash her hair, but her hallucinations and insomnia improved and she was no longer required to take sleeping medication and/or antipsychotic medication.

### Case 2

The patient was a 74-year-old female; 152.9 cm in height and 56.0 kg in weight at the time of her initial examination; a housewife; and described as gentle in her personality. Her chief complaints were lack of motivation, irritability, loss of appetite, and weight loss. Her medical history included hypertension and dyslipidemia at the age of 68, and she had undergone a colectomy at the age of 73 in January X−1. She had no remarkable family medical history, but her husband had Lewy body dementia and was in need of long-term care. Regarding the history of here present illness, she came to our clinic in December X−1 for abnormal behavior based on amnesia and paranoia, such as buying too many vegetables that she could not use, rotting them in the refrigerator, being unable to clean her room, and repeatedly washing her clothes while saying “my husband is dirty.” She had high neuropsychological test scores of 28 on the MMSE and 16 on the FAB; and her ADLs were not abnormally rated low, as indicated by scores of 33 on the DASC-21, 7 on the Lawton IADL scale, and 2 on the level of support required. Neurological examination showed no tendon reflex, left-right hyperactivity, or pathological reflex; and no atrophy was observed in the medial temporal lobe on magnetic resonance imaging (MRI) with coronal transection imaging. However, cerebral blood flow SPECT showed significant blood flow reduction in the anterior part of the bilateral precuneus, which led to the diagnosis of Alzheimer's disease.

She was attending local clinics for internal medicine and orthopedic surgery, but her conditions were poorly controlled, with polypharmacy and a diastolic blood pressure of over 100 mmHg. In our hospital, the patient was not treated with anti-dementia medication and adjustments to her medication regimen were initiated. She was also diagnosed with colorectal cancer and underwent a colectomy in January X. After discharge from the hospital, she weighed 50.6 kg, but her weight increased to 54.4 kg in July X with repeated dietary and nutritional guidance aimed at improving the breakfast menu with a focus on increasing energy levels. However, in September X, depressive symptoms such as “feeling lightheaded and agitated” became noticeable, with frequent missed meals and a decrease in the amount of food intake, which decreased her weight by 1.1 to 53.3 kg in 2 months. Muscle mass measured by a body composition analyzer (InBody 270) using bioelectrical impedance analysis was 32.6 kg. The weight after removing the body fluid weight (24.5 kg) from the total body weight (53.3 kg) was 28.8 kg. Since September X, she started taking 3.75 g of Ninjin'yoeito once before bedtime for her loss of appetite and weight loss.

In November X+1, her appetite improved and she weighed 56.7 kg, with an increase of 3.4 kg. Her muscle mass was 33.0 kg with an increase of 0.4 kg. The weight after removing the body fluid weight was increased by 3.9 kg (56.7–24.0 kg = 32.7 kg).

### Case 3

The patient was an 81-year-old male; 166.0 cm in height and 46.8 kg in weight at the time of his initial examination; running a restaurant business; and described as slovenly in his personality. His chief complaints were memory loss and weight loss, and he had a medical history of hypertension and reflux esophagitis. As to the history of the present illness, he had been suffering from memory loss since around November X−1. He had been treated at a local doctor's clinic for hypertension, but his forgetfulness worsened, as he was asking the same questions over and over again, unable to tell the date of the day, and unable to take his medication, and therefore he came to our clinic in September X. He was diagnosed with Alzheimer's disease, which was already several years advanced, with impairment in recent memory, semantic memory, executive function, and frontal lobe function shown by the scores of 11 on the Hasegawa's dementia scale, 18 on the MMSE, 10 on the FAB, and 10 on the clock-drawing test (CDT), as well as atrophy of the frontal and medial temporal lobes observed on MRI images. In September X, he complained that he was unable to stop drinking and was not eating, and he weighed 46.8 kg, with a decrease of 1.5 kg in 2 months. His muscle mass measured by a body composition analyzer (InBody 270) using bioelectrical impedance analysis was 34.3 kg. The weight after removing the body fluid weight (21.7 kg) from the total body weight (46.8 kg) was 25.1 kg. Since September X, he started taking Ninjin'yoeito 3.75 g once before bedtime.

In November X+1, improvement in his appetite was observed. His weight was 49.3 kg, showing an increase of 2.5 kg. His muscle mass was 36.7 kg, showing an increase of 2.4 kg. The weight after removing the body fluid weight was 49.3–23.2 kg = 26.1 kg, showing an increase of 1.0 kg.

## Discussion

**Figure 1:**
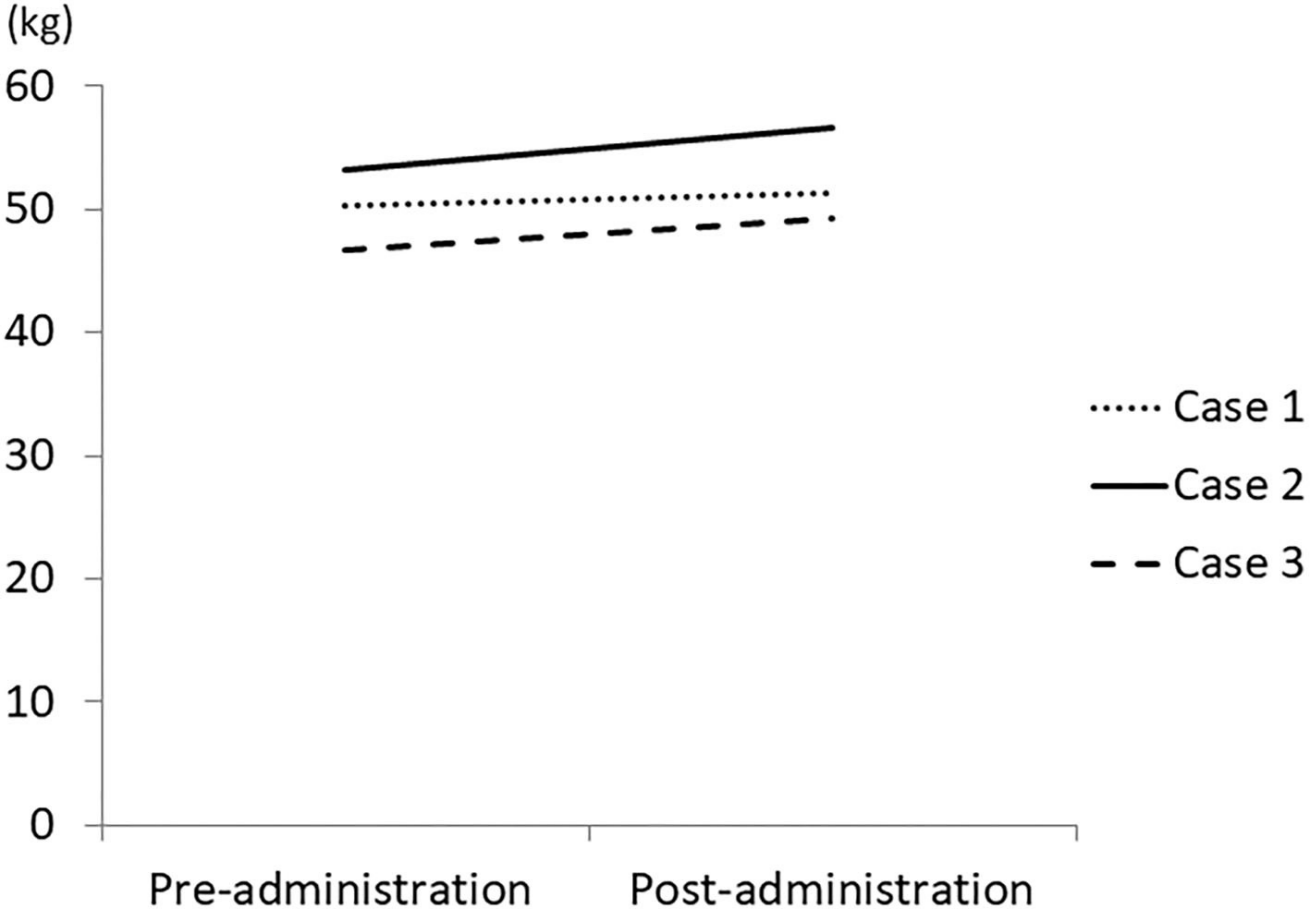
Change in body weight by treatment with Ninjin'yoeito. Three patients who had weight loss of more than 1 kg in 2 months were treated with Ninjin'yoeito and their weight was measured using a body composition analyzer.

**Figure 2:**
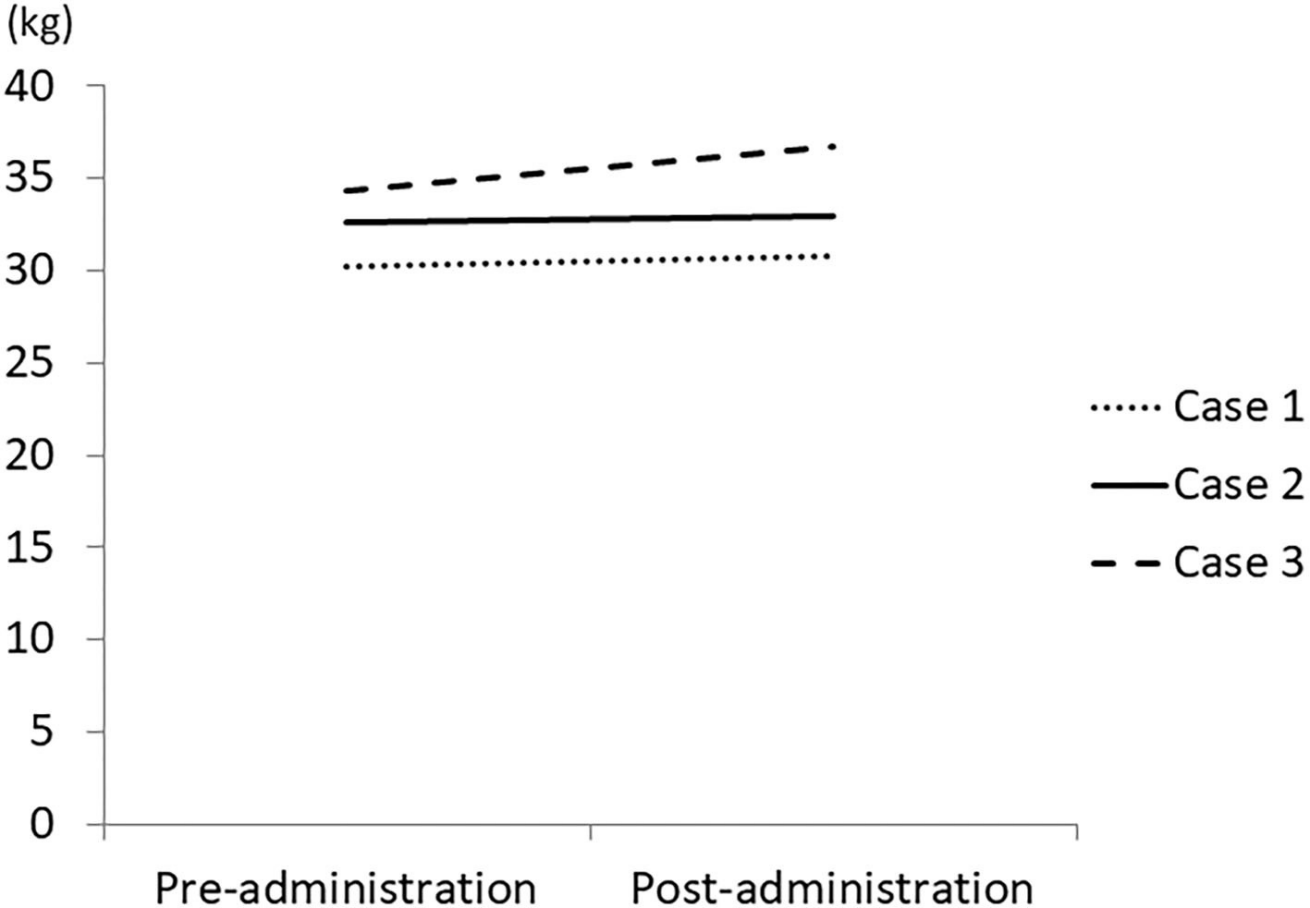
Change in muscle mass by treatment with Ninjin'yoeito. Three patients who had weight loss of more than 1 kg in 2 months were treated with Ninjin'yoeito and their muscle mass was measured using a body composition analyzer.

In this study, 3 patients with dementia who complained of loss of appetite and had a weight loss of more than 1 kg in 2 months were treated with 3.75 g/day of Ninjin'yoeito, after which appetite, body weight, muscle mass, and body fluid loss were increased in all patients (Figures 1, 2).

In the all 3 patients reported in this study, weight gain was achieved beginning around 3 months after initiating the treatment, suggesting that factors other than dementia, such as alcohol consumption, affected nutritional management and delayed the onset of effect.

The mechanism of loss of appetite involves not only organic and functional gastrointestinal lesions, but also central nervous system structures such as the frontal cortex, gustatory and olfactory centers, and the brainstem; and the involvement of the hormones ghrelin and orexin has received particular attention (4). In basic research, it has been reported that Ninjin'yoeito activates both ghrelin-responsive and non-responsive neuropeptide Y (NPY) neurons in the feeding center (5) and promotes ghrelin secretion by Citri Unshiu Pericarpium (6), which may have contributed to the improvement of appetite by Ninjin'yoeito in this study.

Although there was no significant increase in the amount of body fat in the patients reported in this study, I have had patients in the past who have gained weight due to an increase in body fat. However, ghrelin is said to maintain the homeostasis of the organism not only by stimulating feeding, but also as a result of fat accumulation and growth hormone (GH)-induced anabolic effects. Thus, the increase in fat induced by the administration of Ninjin'yoeito may represent a recovery from ghrelin-mediated impaired energy metabolism.

In this study, all 3 patients showed increases in muscle mass, but all had been taking the product for a relatively long period of time, between 9 and 13 months. In a previous report, it required 6 months to improve grip strength in elderly people over 65 years of age after administration of Ninjin'yoeito (7). On the other hand, in basic research, it has been confirmed that administration of Ninjin'yoeito suppresses food intake reduction and weight loss and reduces atrophy of the gastrocnemius and soleus muscles in klotho mice, which are a senescence-accelerated strain. The same study also reported that Ninjin'yoeito activated 4E-BP1, which is involved in protein synthesis, and inhibited the expression of atrogin-1 and LC3-II, which are involved in muscle proteolysis (8). The increase in muscle mass shown in this study may be attributed to the anorexia-reducing effect of Ninjin'yoeito, as well as the promotion of muscle protein synthesis. These findings suggest that Ninjin'yoeito may improve not only loss of appetite and weight loss, but also muscle weakness, which can significantly affect activity, when it is taken for some long periods of time.

A limitation of this study is that the possibility of measurement error due to sweating and salivation cannot be eliminated in the bioelectrical impedance analysis used to measure body composition. In order to confirm the findings of this study, it is necessary to conduct case-series studies with larger sample sizes and to establish measurement conditions.

## Conclusion

There is a close relationship between dementia and physical frailty, such as weight loss and muscle weakness. Therefore, our hospital focuses not only on the administration of anti-dementia drugs in the treatment of dementia, but also on lifestyle-related diseases and nutritional management.

In this study, patients with dementia who complained of loss of appetite and weight loss were administered Ninjin'yoeito, Japanese herbal medicine (Kampo), which resulted in increases in body weight and muscle mass. Ninjin'yoeito was found to be a potentially effective treatment option for physical frailty in patients with dementia.

## Data Availability Statement

The raw data supporting the conclusions of this article will be made available by the authors, without undue reservation.

## Ethics Statement

Ethical review and approval was not required for the study on human participants in accordance with the local legislation and institutional requirements. The patients/participants provided their written informed consent to participate in this study. Written informed consent was obtained from the participant for the publication of any potentially identifiable images or data included in this article.

## Author Contributions

YM and IM conducted the study. YM wrote the manuscript.

## Conflict of Interest

The authors declare that the research was conducted in the absence of any commercial or financial relationships that could be construed as a potential conflict of interest.
